# Evaluation of SARS-CoV-2 Detection Systems Using Clinical Samples and Standard Material: A Comparative Study

**DOI:** 10.3390/diagnostics13122046

**Published:** 2023-06-13

**Authors:** Sunggyun Park, Do-Hoon Kim

**Affiliations:** Departments of Laboratory Medicine, Keimyung University School of Medicine, Daegu 42601, Republic of Korea; nosdolu2@gmail.com

**Keywords:** COVID-19, SARS-CoV-2, limit of detection

## Abstract

Due to the decreasing trends in daily confirmed COVID-19 cases and daily confirmed tests, there is a need for a new testing system capable of quickly and efficiently testing small amounts of samples. Therefore, we compared and evaluated the testing performance of the Aptima SARS-CoV-2 assay, an automated testing system that allows continuous loading of samples, and the Real-Q Direct SARS-CoV-2 detection kit that is currently being used in our laboratory. We compared the results of the two testing systems using 259 residual individual nasopharyngeal specimens and 91 residual pooled nasopharyngeal specimens that were submitted for COVID-19 testing in January and February 2023. The 95% limit of detection (LoD) for the Aptima SARS-CoV-2 assay determined using reference material for SARS-CoV-2 nucleic acid was confirmed to be 17.793 copies/mL, while the LoD for the Real-Q Direct SARS-CoV-2 detection kit was determined to be 131.842 copies/mL for the RdRP gene and 241.77 copies/mL for the E gene. The comparative study using clinical specimens showed almost perfect agreement. Our data showed that the Aptima SARS-CoV-2 assay has a very low LoD. In addition, the Aptima SARS-CoV-2 assay and Real-Q Direct detection kit have comparable clinical performance for SARS-CoV-2 for individual and pooled samples.

## 1. Introduction

Coronavirus disease 2019 (COVID-19) spread rapidly worldwide and was declared a pandemic by the World Health Organization (WHO) on 11 March 2020 [1]. As of March 2023, the cumulative number of confirmed COVID-19 cases in South Korea is approximately 30.6 million, and more than 9000 new cases are still reported on average per day [2]. However, the daily number of confirmed cases of COVID-19 detected through the severe acute respiratory syndrome coronavirus 2 (SARS-CoV-2) polymerase chain reaction (PCR) test has been consistently decreasing. Therefore, the introduction of a new method of conducting tests is necessary, which would involve the rapid processing of individual samples, rather than the previous method of handling large numbers of samples at once, to report results more efficiently.

The Aptima SARS-CoV-2 assay (Hologic, Bedford, MA, USA) is a transcription-mediated amplification (TMA) assay that uses an automated system with a turnaround time of 3.5 h, and can provide random access for reporting results of up to 60 samples per hour continuously thereafter [3]. Clinical comparative data have been obtained for the Aptima SARS-CoV-2 assay [3,4,5,6,7,8]), but to our knowledge, there have been no studies comparing the Aptima SARS-CoV-2 assay and the Real-Q Direct SARS-CoV-2 detection kit (Biosewoom, Seoul, Republic of Korea), which is currently used in our institution, with the same clinical samples. There have also been no studies of the analytical performance of the Real-Q Direct SARS-CoV-2 detection kit using reference materials.

Therefore, this study was performed to compare and evaluate the performance of the Real-Q Direct SARS-CoV-2 detection kit and the Aptima SARS-CoV-2 assay using clinical samples. We also assessed the analytical sensitivity of each assay using reference materials for the entire SARS-CoV-2 genome.

## 2. Materials and Methods

### 2.1. Sample Collection

**Figure 1:**
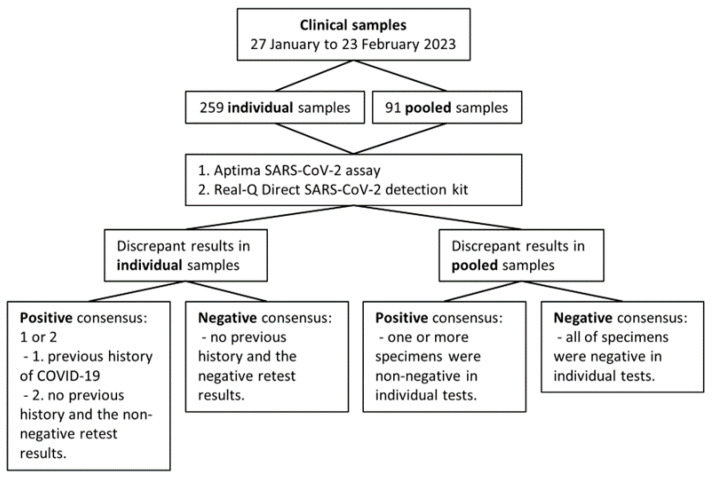
Flowchart for this study, which compares the performance of two types of SARS-CoV-2 real-time PCR assays using clinical samples.

From 27 January to 23 February 2023, a total of 259 individual nasopharyngeal swabs were collected and stored in FA Transport Medium (HLB Healthcare, Sejong, Republic of Korea) at our laboratory for COVID-19 confirmation tests. During the same period, 91 pooled samples consisting of 2–5 individual nasopharyngeal swabs were also collected (Figure 1). This study was approved by the Institutional Review Board of Dongsan Medical Center (DSMC 2023-03-040).

### 2.2. Aptima SARS-CoV-2 Assay for Individual Testing

All tests were conducted according to the respective manufacturer’s instructions. Briefly, 500 μL of transport medium containing nasopharyngeal swab was transferred to a lysis tube containing 710 μL of lysis buffer, which was then directly loaded onto a Panther instrument (Hologic). Each reaction used 460 μL from this lysis tube. Nucleic acids were purified using capture oligonucleotides and a magnetic field. Following amplification, chemiluminescent probes hybridized to the amplicon and emitted light, which was measured using a luminometer and reported in relative light units (RLU). The results of the assay were recorded as positive or negative based on a cutoff RLU value of 600.

### 2.3. Assay Using Real-Q Direct SARS-CoV-2 Detection Kit for Individual Testing

Total nucleic acid from the nasopharyngeal swab in the transport medium was extracted using a Real-prep Viral DNA/RNA Kit (Biosewoom). Nucleic acid was extracted from 200 μL of transport medium containing the nasopharyngeal swab, yielding approximately 60 μL of nucleic acid. A Real-Q Direct SARS-CoV-2 detection kit was used for amplification. This kit consists of a predispensed strip containing a PCR mixture, probe and primer mixture, and enzyme mixture. Briefly, 5 μL of extracted nucleic acid was dispensed into each strip tube and transferred to the CFX96 real-time PCR detection system (Bio-Rad, Richmond, CA, USA) for amplification with the following conditions: 1 cycle of 10 min at 50 °C, 1 cycle of 3 min at 95 °C, 3 cycles of 95 °C for 1 s and 62 °C for 20 s, and 40 cycles of 95 °C for 1 s and 62 °C for 30 s. The Real-Q Direct SARS-CoV-2 detection kit targets two unique genes in the SARS-CoV-2 viral genome (RdRP and E), and the results are interpreted based on the cycle threshold (Ct) value of 38 for each gene. The Ct value of both target genes should be <38 for a positive result, and if only one of the genes has a Ct value < 38 it is recorded as an indeterminate result. Both positive and indeterminate results were defined as non-negative results. Amplification was performed for all batches including a positive control provided by the manufacturer and a negative control using sterile and DNase/RNase-free water.

### 2.4. Pooled Test

Multiple specimens (2–5) were combined to create a single pooled transport medium. For each specimen, 200 μL of the transport medium containing the nasopharyngeal swab was transferred to a sterile conical tube. Then, 500 μL of pooled transport medium was used for the Aptima SARS-CoV-2 assay, and nucleic acid was extracted from 200 μL and used for Real-Q Direct SARS-CoV-2 detection kit assay.

### 2.5. Analytical Sensitivity

The limit of detection (LoD) was determined using AccuPlex™ SARS-CoV-2 Molecular Controls Kit—Full Genome (catalog no. 0505-0159; Seracare, Milford, MA, USA), which was provided at a concentration of 5000 copies/mL. The following serial dilutions were prepared (copies/mL): 500, 250, 125, 62.5, 31.25, 15.625, and 7.8125 for the Aptima SARS-CoV-2 assay; 2500, 1250, 625, 312.5, 156.25, 78.125, and 37.0625 for the Real-Q Direct SARS-CoV-2 detection kit. For dilution, the lysis buffer provided by the manufacturer of the Aptima SARS-CoV-2 assay was used for the Aptima SARS-CoV-2 assay, while negative material containing the human RNase P gene included in the AccuPlex™ SARS-CoV-2 Molecular Controls kit was used for the Real-Q Direct SARS-CoV-2 detection kit. We initially conducted 10 repetitions for each concentration. We intended to determine a more accurate limit of detection (LOD) by conducting an additional 10 repetitions specifically for the concentration with the lowest level where all 10 initial repetitions showed positive results. Accordingly, for the Aptima SARS-CoV-2 assay, samples with concentrations of 31.25 copies/mL, and for the Real-Q Direct SARS-CoV-2 detection kit, samples with concentrations of 156.25 copies/mL were subjected to 20 repetitions each.

### 2.6. Statistical Analysis

The LoD was calculated by probit analysis by measuring the diluted materials. The positive percent agreement (PPA), negative percent agreement (NPA), positive rate, kappa, and two-sided (upper/lower) 95% confidence interval (CI) were calculated for comparative study with clinical nasopharyngeal swabs. McNemar’s chi-square test was also performed. All statistical analyses were performed using R (version 4.2.2; R Foundation for Statistical Computing, Vienna, Austria) and RStudio (Desktop version 2022.07.2+576; Posit, Boston, MA, USA).

In the comparative study with clinical individual nasopharyngeal swabs, in cases where the results of the two assays were discrepant (one non-negative and the other negative), a consensus was reached based on the previous history of COVID-19 and the retest results (Figure 1). We checked the National Notifiable Disease Surveillance System of South Korea to confirm cases of the patient’s previous history of COVID-19. The criteria for a positive consensus result for individual tests were a previous history of COVID-19 or no previous history of COVID-19, but the sample was retested with the assay that showed a non-negative result and yielded a consistent non-negative result. For the comparative study with pooled samples, a positive consensus result was defined as a case where one or more specimens were non-negative in individual testing performed with Aptima SARS-CoV-2 assay.

## 3. Results

### 3.1. Analytical Sensitivity

**Figure 2:**
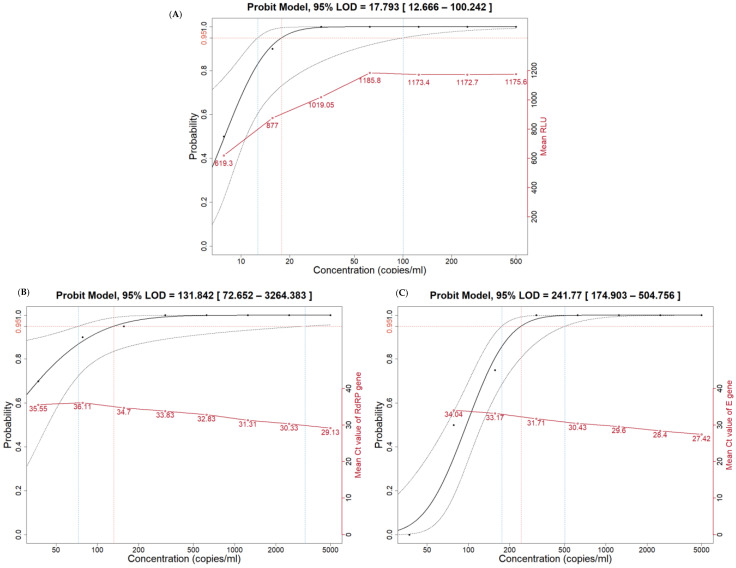
The limit of detection (LoD) of 95% via probit analysis for each assay, including the Aptima SARS-CoV-2 assay (**A**), RdRP gene (**B**), and E gene (**C**) with the Real-Q Direct SARS-CoV-2 detection kit using SARS-CoV-2 nucleic acid material. The orange horizontal dotted line in each plot indicates the positive result probability of 0.95 for each concentration of SARS-CoV-2 nucleic acid material. The orange vertical dotted line in each plot indicates the 95% LoD via probit analysis. The blue vertical dotted line in each plot indicates the 95% confidence interval of LoD. Black dots represent the positive rate of each concentration of SARS-CoV-2 nucleic acid material. Open red circles represent the mean RLU value (**A**) or mean Ct value (**A**,**B**) of each assay.

The LoDs for each assay using SARS-CoV-2 nucleic acid were as follows: 17.793 copies/mL for the Aptima SARS-CoV-2 assay; 131.842 copies/mL for the RdRP gene; and 241.77 copies/mL for the E gene in the Real-Q Direct SARS-CoV-2 detection kit. The 95% CIs for the LoD values and the mean RLU and Ct value of each assay are shown in Figure 2.

### 3.2. Clinical Performance of Individual Testing

Using the results obtained with the Real-Q Direct SARS-CoV-2 detection kit as a reference, the PPA for the Aptima SARS-CoV-2 assay was approximately 89.8% for 259 nasopharyngeal swab specimens (Table 1). The agreement between the two tests had a Cohen’s kappa coefficient of 0.886 indicating almost perfect agreement. The results of the two assays were also compared using McNemar’s chi-square test, which did not show a significant difference.

There were nine cases of discrepancies between the two assays, five of which were non-negative in the Real-Q Direct SARS-CoV-2 detection kit and negative in the Aptima SARS-CoV-2 assay, and four were negative in the Real-Q Direct SARS-CoV-2 detection kit but positive in the Aptima SARS-CoV-2 assay (Table 2).

Based on a comparison with the consensus result determined from the previous history of COVID-19 and retest results, the Aptima SARS-CoV-2 assay was estimated to have two false positives and three false negative cases, while the Real-Q Direct SARS-CoV-2 detection kit was estimated to have two false positives and two false negative cases. The Ct values and RLU for each discrepant case are shown in Table 2.

### 3.3. Clinical Performance of Pooled Testing

Using the results from the Real-Q Direct SARS-CoV-2 detection kit as the reference, the PPA for the Aptima SARS-CoV-2 assay was approximately 90.0% for 91 pooled samples consisting of 2–5 individual samples (Table 1). The tests showed almost perfect agreement with Cohen’s kappa coefficient of 0.872. McNemar’s chi-square test also showed no significant difference in results between the two assays.

There were four discrepant cases between the two assays, consisting of two in which the results were non-negative with the Real-Q Direct SARS-CoV-2 detection kit but negative with the Aptima SARS-CoV-2 assay, and two cases with the opposite results (Table 3). The Ct values and RLU for each discrepant case are presented in Table 3.

## 4. Discussion

Since COVID-19 was first declared a pandemic in 2020, the daily number of new COVID-19 cases in South Korea has fluctuated between increasing and decreasing but has been consistently decreasing since the beginning of 2023 [2]. The daily number of COVID-19 tests for confirmation has also been decreasing in line with the decreasing trend of new cases. The average daily number of COVID-19 confirmation tests conducted at our laboratory has also been consistently decreasing, with 26 individual tests and 20 pooled tests conducted per day in January and February 2023 compared to 84 individual tests and 23 pooled tests per day in 2022. Accordingly, there is increasing demand for automated testing systems that can efficiently process individual samples rather than relying on batch testing systems for handling large volumes of samples.

The Aptima SARS-CoV-2 assay is a nucleic acid amplification test based on target capture and TMA technologies. It is a fully automated system capable of loading consecutive samples in batches of five. This TMA method targets two unique regions of the SARS-CoV-2 ORF-1ab gene and proceeds in a one-step process under isothermal conditions. Previous studies reported the low LoD of the Aptima SARS-CoV-2 assay, ranging from 62.5 to 870 copies/mL [3,5,6]. Based on this low LoD, Kimberly et al. used the assay as a COVID-19 screening tool using 10/1 pooled samples [9]. In our study, the LoD of the Aptima SARS-CoV-2 assay was found to be even lower than in previous reports at 18.2 copies/mL. Here, we conducted a probit analysis to evaluate the 95% LoD of the Aptima SARS-CoV-2 assay. To achieve a narrower confidence interval than previous studies, we performed serial dilutions and included even lower concentrations of nucleic acids. The results confirmed a significantly lower LoD than in previous studies.

The Real-Q Direct SARS-CoV-2 detection kit is a premixed PCR reagent that improves the analytical sensitivity and usability of the Real-Q nCoV-2019 detection kit from the same manufacturer. The analytical sensitivity and comparative studies with clinical samples have been reported for the Real-Q nCoV-2019 detection kit [10,11]. However, there have been no reports of performance verification of the Real-Q Direct SARS-CoV-2 detection kit. The LoDs of the Real-Q Direct SARS-CoV-2 detection kit were determined in this study as 131.8 copies/mL for the RdRP gene and 241.8 copies/mL for the E gene. The LoD of the Real-Q Direct SARS-CoV-2 detection kit confirmed in this study showed sufficiently good analytical sensitivity with a lower value than reported for the Real-Q nCoV-2019 detection kit (4030 copies/mL) [10].

The concordance between the Aptima SARS-CoV-2 assay and the Real-Q Direct SARS-CoV-2 detection kit was confirmed to be very high using individual test results from 259 clinical nasopharyngeal swabs.

Of the nine discrepant results, four false positives were confirmed to be negative through retesting, suggesting the possibility of nucleic acid or sample contamination or nonspecific amplification. Cases #I1 and #I6 with high Ct values near 35 or low RLU values were retested (Table 2). However, cases #I5 and #I8 that showed low Ct values and high RLU values tested negative upon retesting, and therefore the possibility of a major error, including sample mix-up, could not be excluded.

All three cases (#I4, #I7, and #I9) that showed false positive results on the Aptima SARS-CoV-2 assay with even lower LoD were confirmed to have low levels of SARS-CoV-2 nucleic acid with high Ct values close to 35 using the Real-Q Direct SARS-CoV-2 detection kit, suggesting a limitation of the Aptima SARS-CoV-2 assay that does not involve extraction processes other than the use of lysis buffer.

The concordance between the two assays was also very high in the pooled test results using 91 pooled samples. Of the four discrepant results, the false positive case with the Aptima SARS-CoV-2 assay (#P1) showed a high RLU value but tested negative in all individual tests, and the possibility of sample contamination or nonspecific amplification could not be excluded. The false positive case with the Real-Q Direct SARS-CoV-2 detection kit (#P3) showed an indeterminate result with only E gene amplification and was subjected to retesting, which confirmed negative results in all individual tests.

The false positive case with the Aptima SARS-CoV-2 assay showed RLU value below the cutoff but above 350, and was subjected to retesting, which confirmed positive results in one of the individual tests. La et al. suggested using a secondary method to confirm cases with RLU values above 350 in the Aptima SARS-CoV-2 assay to improve the detection of low viral loads [6], and this suggestion was supported by case #P4 in the present study.

This study has several limitations. Due to the low positive rate during the study period, we were unable to include sufficient positive samples with a relatively even distribution of Ct values. Moreover, due to the insufficient number of positive samples, it was not possible to deeply investigate how the Aptima SARS-CoV-2 assay and Real-Q Direct detection kit can complement each other in this study. However, the difference in performance between these two assay reagents underscores the importance of using multiple reagents from different manufacturers for confirmation purposes to ensure accurate testing. This approach is crucial for effective infection control and prompt and accurate diagnosis of patients. In addition, in cases where the two assays showed discrepant results, we were unable to confirm the results using a method with even higher analytical sensitivity, such as digital droplet/partition PCR. The use of only one quantified standard for LoD establishment could also represent a limitation.

In conclusion, our data showed that the Aptima SARS-CoV-2 assay has very low LoD. In addition, the Aptima SARS-CoV-2 assay and Real-Q Direct detection kit have comparable clinical performance for the detection of SARS-CoV-2 in nasopharyngeal swabs on individual and pooled testing. These performance characteristics should be taken into consideration when making testing platform decisions.

## Figures and Tables

**Table 1:** Clinical performance comparison of two assays for 259 individual and 91 pooled nasopharyngeal swabs.

Aptima SARS-CoV-2 Assay	Real-Q Direct SARS-CoV-2 Detection Kit		Kappa (95% CI)	McNemar’s (*p*-Value)	PPA (%) (95% CI)	NPA (%) (95% CI)
	Non-Negative	Negative				
Individual test						
Positive	44	4	0.886 (0.813–0.959)	1	89.8 (78.2–95.6)	98.1 (95.2–99.3)
Negative	5	206				
Pooled test						
Positive	18	2	0.872 (0.749–0.994)	1	90.0 (69.9–97.2)	97.2 (90.3–99.2)
Negative	2	69				

Abbreviations: CI, confidence interval; NPA, negative percent agreement; PPA, positive percent agreement.

**Table 2:** Details of discordant results of individual tests.

Sample ID	Real-Q Direct SARS-CoV-2 Detection Kit				Aptima SARS-CoV-2 Assay		Previous COVID-19	Retest (RLU or Ct *E*-*RdRP*) *	Consensus Result
	*E* (Ct)	*RdRP* (Ct)	IC (Ct)	Interpretation	RLU	Interpretation			
I1	34.74	37.52	24.03	Positive	252	Negative	No	Negative (NA-NA)	FP
I2	NA	NA	22.06	Negative	687	Positive	Yes		FN
I3	NA	NA	22.58	Negative	774	Positive	Yes		FN
I4	33.84	34.91	22.04	Positive	262	Negative	Yes		FN
I5	25.2	24.97	26.17	Positive	259	Negative	No	Negative (NA-NA)	FP
I6	NA	NA	25.62	Negative	699	Positive	No	Negative (263)	FP
I7	35.18	NA	24.34	Indeterminate	257	Negative	No	Positive (33.72–34.11)	FN
I8	NA	NA	23.44	Negative	1146	Positive	No	Negative (249)	FP
I9	35.57	35.32	22.39	Positive	267	Negative	Yes		FN

Abbreviations: Ct, cycle threshold; FN, false negative; FP, false positive; IC, internal control; NA, not amplified; RLU, relative light units. * Retest was performed using the assay that yielded a non-negative result in the initial test.

**Table 3:** Details of discordant results of pooled tests.

Sample ID		Real-Q Direct SARS-CoV-2 Detection Kit				Aptima SARS-CoV-2 Assay		Result of Split (RLU) *	Consensus Result
		*E* (Ct)	*RdRP* (Ct)	IC (Ct)	Interpretation	RLU	Interpretation		
P1	1p-1	NA	NA	23.35	Negative	1178	Positive	Negative	FP
	1p-2							Negative	
	1p-3							Negative	
	1p-4							Negative	
	1p-5							Negative	
P2	2p-1	NA	NA	24.78	Negative	895	Positive	Negative	FN
	2p-2							Negative	
	2p-3							Negative	
	2p-4							Positive (1130)	
	2p-5							Negative	
P3	3p-1	35.6	NA	23.95	Indeterminate	273	Negative	Negative	FP
	3p-2							Negative	
	3p-3							Negative	
	3p-4							Negative	
	3p-5							Negative	
P4	4p-1	33.17	35.11	21.97	Positive	460	Negative	Positive (1154)	FN
	4p-2							Negative	
	4p-3							Negative	
	4p-4							Negative	
	4p-5							Negative	

Abbreviations: Ct, cycle threshold; FN, false negative; FP, false positive; IC, internal control; NA, not amplified; RLU, relative light units. * Individual tests were performed using the Aptima SARS-CoV-2 assay.

## Data Availability

The data presented in this study are available on request from the corresponding author.
